# Clinical characteristics, prognostic factors, and long‐term outcomes associated with epithelial malignancies of the thymus: A 20‐year single‐institution experience

**DOI:** 10.1002/cnr2.1750

**Published:** 2022-11-12

**Authors:** Ellery Altshuler, Akash Mathavan, Akshay Mathavan, Urszula Krekora, Mohit Mathavan, Keegan Hones, Karen Daily

**Affiliations:** ^1^ Department of Internal Medicine University of Florida Gainesville Florida USA; ^2^ College of Medicine University of Florida Gainesville Florida USA; ^3^ College of Medicine University of Central Florida Orlando Florida USA; ^4^ Department of Internal Medicine St George's University School of Medicine Great River New York USA; ^5^ Division of Hematology and Oncology University of Florida Gainesville Florida USA

**Keywords:** thymic carcinoma, thymic epithelial tumors, thymoma

## Abstract

**Background:**

Thymic epithelial tumors are rare and include thymomas and thymic carcinomas. There is scarce literature characterizing prognostic factors and long‐term outcomes in these tumors.

**Aims:**

This review aims to describe disease features of thymomas and thymic carcinomas and to report clinical differences among thymoma histological subtypes.

**Methods and Results:**

A retrospective chart review was performed at the University of Florida Shands Hospital, a tertiary care academic medical center in Gainesville, Florida, USA. The review included clinical data of adults with thymic epithelial tumors diagnosed between 2001 and 2021. Significant associations among demographics, histology, stage, and outcomes were investigated. Thymoma subgroup analysis was performed using histological subtype and sex. Forty patients with thymoma and seven patients with thymic carcinoma were included in the final analysis. Among those with thymomas, patients with subtype B1, B2, or B3 tumors were younger, had larger tumors, and presented with higher stage disease when compared to those with subtypes A or AB. Tumor recurrence was most common in subtype B2 and B3 tumors (50.0% and 16.7% vs. 0%; *p* < .01). However, there was no significant difference in overall survival between histologic subtypes. Compared to females, males with thymomas had superior overall survival (103.0 vs. 62.9 months; *p* = .021) despite presenting with larger tumors (9.8 vs. 5.8 cm; *p* = .041). Concomitant myasthenia gravis was associated with increased recurrence but not worsened mortality. Compared to thymomas, patients with thymic carcinoma presented with higher‐stage disease and had poorer 5‐year survival (50.0% vs. 93.1%; *p* < .01).

**Conclusion:**

This study affirmed pathologic stage and resectability as prognostic factors for thymic epithelial tumors. New findings include inferior overall survival in female patients and higher recurrence rates in those with thymomas and concomitant myasthenia gravis.

## 1 INTRODUCTION

Thymic epithelial tumors encompass thymomas and thymic carcinomas. These tumors comprise roughly one‐fifth of mediastinal masses and most commonly present between the ages of 40 and 60.^1^ Thymomas are generally indolent, albeit malignant, neoplasms with a tendency for local spread and several immunological manifestations such as myasthenia gravis or pure red cell aplasia.^2^ Other associated autoimmune pathologies include inflammatory myopathies, systemic lupus erythematosus, and autoimmune bullous dermatoses‐autoimmune blistering diseases (e.g., pemphigus vulgaris, paraneoplastic pemphigus, bullous pemphigoid).

Thymic epithelial tumors are histologically arranged according to the World Health Organization (WHO) classification scheme, which identifies type A and B tumors, defined by the presence of spindle‐oval versus stellate‐polygonal epithelial cells, respectively. These tumor types are further divided into subtypes: A, AB, B1, B2, and B3 epithelial thymomas based on organotypical features such as composition of tissue and pattern of growth.^3^ Thymic carcinomas (WHO type C) are more aggressive, high‐grade cancers identified by cellular atypia and infiltrative growth patterns on histology that present with obstructive symptoms due to tumor extension and metastasis.

Thymic epithelial tumors are staged according to the tumor, node, and metastasis system of the American Joint Committee on Cancer (AJCC)/Union for International Cancer Control (UICC); however, the alternative Masaoka system stages thymic epithelial tumors by degree of macroscopic and microscopic tissue invasion and has been widely used in descriptive and investigative studies.^4^, ^5^

With an incidence of 1–2 cases per 100 000 person‐years, thymic cancers are exceedingly rare with exceptionally diverse presentations.^1^ Although WHO classification lends credible prognostic significance, as subtype A‐B2 tumors are often more localized on presentation than subtype B3‐C tumors, features such as patient characteristics (e.g., age of onset, gender predilection) and the combined effect of stage and histologic classification on both prognosis and recurrence are less well described. There is an overall paucity of data on clinical characteristics, prognostic indicators, and management. This institutional retrospective cohort study aimed to address this deficit by describing disease characteristics and long‐term outcomes in patients with thymomas and thymic carcinomas.

## 2 METHODS

### 2.1 Database and study population

Approval for this study was obtained from the University of Florida Institutional Review Board (IRB202102647). Patients were identified using the University of Florida Shands Hospital (UFHSH) integrated data repository. Patients were included if they had biopsy‐confirmed thymic epithelial malignancy treated between January 1, 2001, and January 1, 2021. Patients were then categorized according to whether they had a diagnosis of thymoma or thymic carcinoma. Thymomas were histologically classified according to the WHO scheme; this includes subtypes A (medullary thymoma), AB (mixed thymoma), B1 (predominantly cortical thymoma), B2 (cortical thymoma), and B3 (well‐differentiated thymic carcinoma), which are based on morphologic similarity to normal thymus tissue and percent of tumor‐invading lymphocytes.^3^ Among patients with thymoma, subgroup analysis was performed based on tumor subtype and sex. Stages were determined using the AJCC Staging, 8th edition.^4^ Baseline demographic, pathologic, and clinical information was also extracted.

### 2.2 Statistical analysis

Statistical analysis was performed with SPSS 28.0.1.0 (142) (IBM Corp). The significance of differences between expected and observed frequencies was assessed with *χ* ^2^, Fisher's exact, and Wilcoxon tests. A 95% confidence interval with a *p* value of less than .05 was used as a cutoff for statistical significance. In addition, survival analysis was conducted using Kaplan Meier curves, log‐rank test, and Cox proportional hazards model. Multivariate analysis was not performed.

## 3 RESULTS

### 3.1 Patient characteristics

A total of 40‐seven patients diagnosed with epithelial thymic malignancies were treated at University of Florida over a 20‐year period. There were 20 patients diagnosed with a thymoma and seven patients diagnosed with thymic carcinoma. Baseline characteristics for these two groups are included in Table 1. There was no difference in median age of patients (53.2 vs. 55.7 years old; *p* = .680) at time of diagnosis for thymoma versus thymic carcinoma. Additionally, no significant difference of incidence based on gender or a history of smoking (55% vs. 57%; *p* = .957) was identified between patients with thymoma versus those with thymic carcinoma. Patients with thymoma were more likely than patients with thymic carcinoma to have a history of autoimmune disease (40% vs. 0%; *p* = .039). The most prevalent autoimmune manifestation was myasthenia gravis; no instances of inflammatory myopathies or autoimmune bullous dermatoses‐autoimmune blistering diseases were noted.

**TABLE 1:** Patient characteristics

	Overall (*n* = 47)	Thymoma (*n* = 40)	Thymic carcinoma (*n* = 7)	*p* value
Age, years (range)	54.0 (18–80)	53.2 (18–76)	55.7 (41–80)	.680
Sex, *n* (%)				
Male	26 (55.3%)	20 (50.0%)	6 (85.7%)	.080
Female	21 (44.7%)	20 (50.0%)	1 (14.3%)	
Race, *n* (%)				
White	30 (63.8%)	26 (65.0%)	4 (57.1%)	.639
Black/African American	12 (25.5%)	9 (22.5%)	3 (42.9%)	
Hispanic/Latino	1 (2.2%)	1 (2.5%)	0 (0%)	
Asian or Pacific Islander	3 (6.4%)	3 (7.5%)	0 (0%)	
Medical history, *n* (%)				
Autoimmune disease	16 (34.0%)	16 (40.0%)	0 (0%)	.039
Myasthenia gravis	14 (29.8%)	14 (35.0%)	0 (0%)	.082
Hypothyroidism	5 (10.6%)	5 (12.5%)	0 (0%)	.352
Secondary malignancy	7 (14.9%)	5 (12.5%)	2 (28.6%)	.271
Red cell aplasia	2 (4.2%)	2 (5.0%)	0 (0%)	.545
Smoking, *n* (%)				
Active	8 (17.0%)	7 (17.5%)	1 (14.3%)	.957
Former	18 (38.3%)	15 (37.5%)	3 (42.9%)	
Never	21 (44.7%)	18 (45.0%)	3 (42.9%)	

### 3.2 Disease characteristics

Tumor characteristics are listed in Table 2. Thymomas were more likely to have been discovered incidentally than thymic carcinomas; however, this result was not statistically significant (42.5% vs. 14.3%; *p* = .157). Furthermore, patients with age greater than 65‐year‐old were more likely to have their tumors discovered incidentally (76.9% vs. 23.5%; *p* < .01). Among symptomatic patients in both groups, dyspnea and chest pain were the most common symptoms (34.0% and 19.1%, respectively). Thymic carcinomas were more likely present at a higher stage (*p* = .024) and to be treated with chemotherapy (85.7% vs. 15.0%; *p* < .01) and radiation therapy (85.7% vs. 27.5%; *p* < .01). Patients with thymoma were more likely to undergo surgical resection via thymectomy (97.5% vs. 71.4%; *p* < .01) and, among patients who underwent surgery, were more likely to have microscopically negative margins (84.6% vs. 0.0%; *p* = <.01). Tumor recurrence was more common in patients with myasthenia gravis (38.5% vs. 10.7%; *p* = .037), higher stage disease (*p* = .026), and positive surgical margins (*p* = .028). Detailed patient characteristics on the seven patients with thymic carcinomas are provided in Table 3.

**TABLE 2:** Disease characteristics and outcomes

	Overall (*n* = 47)	Thymoma (*n* = 40)	Thymic carcinoma (*n* = 7)	*p* value
Disease presentation, *n* (%)				
Incidental discovery	18 (38.3%)	17 (42.5%)	1 (14.3%)	.157
Dyspnea	16 (34.0%)	14 (35.0%)	2 (28.6%)	
Chest pain	9 (19.1%)	7 (17.5%)	2 (28.6%)	
Cough	1 (2.1%)	0 (0%)	1 (14.3%)	
Dysphagia	3 (6.4%)	2 (5.0%)	1 (14.3%)	
Tumor size, cm (range)	7.8 (2.5–9.8)	8.1 (2.5–27.2)	5.8 (2.7–9.8)	.328
Stage (AJCC), *n* (%)				
Stage I	19 (40.4%)	18 (45.0%)	1 (14.3%)	<.01
Stage II	9 (19.1%)	9 (22.5%)	0 (0.0%)	
Stage IIIa	3 (6.4%)	2 (5.0%)	1 (14.3%)	
Stage IIIb	5 (10.6%)	5 (12.5%)	0 (0.0%)	
Stage IVa	6 (12.8%)	4 (10.0%)	2 (28.6%)	
Stage IVb	5 (10.6%)	2 (5.0%)	3 (42.9%)	
Treatment, *n* (%)				
Chemotherapy	12 (25.5%)	6 (15.0%)	6 (85.7%)	<.01
Radiation	17 (36.2%)	11 (27.5%)	6 (85.7%)	<.01
Surgery	44 (93.6%)	39 (97.5%)	5 (71.4%)	<.01
Surgical margins, *n* (%)^a^				
Negative	33 (70.2%)	33 (84.6%)	0 (0%)	<.01
R1 (positive microscopically)	8 (17.0%)	4 (10.3%)	4 (80.0%)	
R2 (positive macroscopically)	3 (6.4%)	2 (5.1%)	1 (20.0%)	
Remission achieved, *n* (%)	36 (76.6%)	32 (80.0%)	4 (57.1%)	.037
Disease recurrence, *n* (%)	8 (17.0%)	7 (17.5%)	1 (14.3%)	.815
Median overall survival, months	72.0	72.5	46.0	.614
2‐year survival, *n* (%)^b^	40 (85.1%)	35 (94.6%)	5 (71.4%)	.051
5‐year survival, *n* (%)^b^	30 (63.8%)	27 (93.1%)	3 (50%)	<.01

^a^ Percentages are provided relative to total number of patients that underwent surgery.

^b^ Percentages are provided relative to total number of patients for whom 2‐ and 5‐year data were available.

**TABLE 3:** Disease characteristics of patients with thymic carcinoma

Pt. no	Sex	Presenting complaint	Histology	IHC	Stage	Treatment	Outcome
1	M	Dyspnea	NOS	CK 5/6, CK 8/18	IVa	XRT, cisplatin + etoposide	Died at 4 months due to disease progression
2	M	Chest pain	Squamous	p63, CD 117	I	Surgery only	Alive at 4 years without recurrence
3	M	Dyspnea	NOS	CAM 5.2, p63	IVb	Surgery, XRT, chemo (platinum‐based)	Alive at 10 years without recurrence
4	M	Dry cough	Squamous	Missing	IVb	XRT, cisplatin + etoposide	Died at 4 months due to disease progression
5	F	Missing	Squamous	Missing	Missing	Surgery, XRT, carboplatin + paclitaxel	Alive at 18 years without recurrence
6	M	Dyspnea	Mixed squamous	p40, p63, CD 117	IIIa	Surgery, XRT, carboplatin, sunitinib, nivolumab, pembrolizumab	Alive at 18 years with recurrence at 9, 15 years
7	M	Incidental	NOS	p40, CK 5/6	IVb	Missing	Died at 18 months due to disease progression

Abbreviations: NOS, not otherwise specified; XRT, radiation therapy.

### 3.3 Thymoma subgroup analysis: histological subtypes

Among patients with thymoma, subtypes included: A (*n* = 3; 7.5%), AB (*n* = 9, 22.5%), B1 (*n* = 10, 25.0%), B2 (*n* = 8, 20.0%), and B3 (*n* = 6, 15.0%), as reported in Table 4. Of note, four verified cases of thymoma did not have a documented histological subtype. Compared to patients with subtype A or AB tumors, patients with B1, B2, or B3 tumors were younger (*p* = .031), had larger tumors (*p* = .012), and higher stage disease (*p* < .01). Tumor recurrence was most common in subtype B2 tumors (*p* < .01) and was not observed in any patients with subtype A, AB, or B1 tumors. There was no significant difference in overall survival in patients with different thymoma subtypes (*p* = .724).

**TABLE 4:** Thymoma subgroup analysis based on histological subtype

	A (*n* = 3)	AB (*n* = 9)	B1 (*n* = 10)	B2 (*n* = 8)	B3 (*n* = 6)	*p* value
Age (years), median	71.0	63.0	43.0	52.0	47.0	.031
Size (cm), median	4.9	4.7	5.4	8.0	9.7	.012
Stage, *n* (%)						
I	1 (33.3%)	6 (66.7%)	6 (60.0%)	4 (50.0%)	0 (0%)	<.01
II	2 (66.7%)	2 (22.2%)	3 (30.0%)	2 (25.0%)	0 (0%)	
IIIa	0 (0%)	1 (11.1%)	0 (0%)	0 (0%)	1 (16.7%)	
IIIb	0 (0%)	0 (0%)	0 (0%)	0 (0%)	3 (50.0%)	
IVa	0 (0%)	0 (0%)	1 (10.0%)	1 (12.5%)	2 (33.3%)	
IVb	0 (0%)	0 (0%)	0 (0%)	1 (12.5%)	0 (0%)	
Treatment, *n* (%)						
Surgery	3 (100%)	9 (100%)	9 (90.0%)	8 (100%)	6 (100%)	.614
Chemotherapy	0 (0%)	0 (0%)	0 (0%)	2 (25.0%)	4 (66.7%)	<.01
Radiation	2 (66.7%)	0 (0%)	0 (0%)	1 (12.5%)	6 (100%)	<.01
Tumor recurrence, *n* (%)	0 (0%)	0 (0%)	0 (0%)	4 (50.0%)	1 (16.7%)	<.01
Overall survival (months), median	77.3	62.6	65.4	95.8	80.4	.724

*Note*: Four individuals of the thymoma subgroup did not have a documented histologic subtype and are not included in the data above.

### 3.4 Thymoma subgroup analysis: male and female sex

No differences were observed between male and female patients with thymoma in age, stage, thymoma subtype, frequency of myasthenia gravis, or rate of complete surgical resection (Table 5). Thymomas in males were larger than those in females (9.8 vs. 5.8 cm; *p* = .041). Overall survival was higher in males than in females (103.0 vs. 62.9 months; *p* = .021).

**TABLE 5:** Thymoma subgroup analysis based on sex

	Male (*n* = 20)	Female (*n* = 20)	*p* value
Age, years, mean (range)	53.1 (34–74)	53.3 (18–76)	.976
Tumor size, cm, mean (range)	9.8 (4.1–27.2)	5.8 (2.5–15.0)	.041
Size ≥5 cm, *n* (%)	15 (75.0%)	6 (30.0%)	.033
Presenting symptoms, *n* (%)			
Incidental discovery	9 (45.0%)	8 (40.0%)	.478
Exertional dyspnea	8 (40.0%)	6 (30.0%)	
Chest pain	3 (15.0%)	4 (20.0%)	
Dysphagia	0 (0.0%)	2 (10.0%)	
Subtype, *n* (%)^a^			
A	0 (0%)	3 (15.0%)	.369
AB	5 (25.0%)	4 (25.0%)	
B1	6 (30.0%)	4 (25.0%)	
B2	5 (25.0%)	3 (15.0%)	
B3	4 (20.0%)	2 (10.0%)	
Stage, *n* (%)			
I	8 (40.0%)	10 (50.0%)	.809
II	6 (30.0%)	3 (15.0%)	
IIIa	0 (0%)	4 (20.0%)	
IIIb	3 (15.0%)	0 (0%)	
IVa	3 (15.0%)	1 (5.0%)	
IVb	0 (0%)	2 (10.0%)	
Surgery, *n* (%)^b^	19 (95.0%)	20 (100%)	.311
R0: Negative margins	16 (84.2%)	17 (85.0%)	.948
R1: Positive macroscopic margins	2 (10.5%)	2 (10.0%)	
R2: Positive macroscopic margins	1 (5.3%)	1 (5.0%)	
Any autoimmune disease, *n* (%)	8 (40.0%)	8 (40.0%)	1.000
Myasthenia gravis	7 (35.0%)	7 (35.0%)	1.000
Overall survival, months, mean/median (range)	103.0/87.5 (21.0–241.0)	62.9/68.5 (19.0–138.2)	.021

^a^ Four individuals of the thymoma subgroup did not have a documented histologic subtype.

^b^ Percentages are provided relative to total number of patients that underwent surgery.

### 3.5 Survival analysis

The average follow‐up time for all patients was 84.8 months. Median survival was 72.5 months in patients with thymoma and 46.0 months in patients with thymic carcinoma (*p* = .630). Kaplan–Meier survival curves for patients with thymoma and thymic carcinoma, as well as survival curves based on patient stage (AJCC Staging) and sex, are provided in Supporting Information. Five‐year survival was higher in patients with thymoma than in patients with thymic carcinoma (93.1% vs. 50.0%; *p* < .01). Undergoing surgical intervention was associated with improved survival (*p* < .01), and higher stage disease was associated with a worse outcome (*p* < .01).

On univariate analysis of patients with thymoma, age at diagnosis, size ≥5 cm, negative surgical margins, and myasthenia gravis were not associated with significantly increased mortality (*p* > 0.25), as reported in Table 6. Female sex and stage IV disease were associated with an increased risk of death; specifically, female sex was associated with significantly worse overall survival when compared to males (62.9 months vs. 103.0 months, *p* = .021; hazard ratio 95% confidence interval 7.357 [1.398–38.706]). Of the seven instances of disease recurrence in the thymoma cohort, four individuals were female.

**TABLE 6:** Univariate survival analysis for patients with thymoma

	Hazard ratio (95% CI)	*p* value
Age at diagnosis	1.021 [0.977–1.068]	.350
Female sex	7.357 [1.398–38.7]	.008
Race, Black; ref: White	0.498 [0.060–4.161]	.520
Size ≥5 cm	1.669 [0.181–15.364]	.636
Symptomatic at presentation	0.301 [0.033–2.712]	.284
Stage IV disease	6.350 [2.004–20.116]	.002
Surgery	0.060 [0.005–0.674]	.002
R0: Negative macroscopic margins	0.965 [0.276–3.380]	.955
Radiation	1.094 [0.269–4.451]	.900
Chemotherapy	1.468 [0.295–7.296]	.639
Smoking history	1.206 [0.323–4.504]	.781
Any autoimmune disease	2.256 [0.555–9.119]	.256
Myasthenia gravis	2.014 [0.537–7.551]	.298

## 4 DISCUSSION

In this institutional retrospective cohort study, we investigated clinical presentation, prognostic factors, and long‐term outcomes of patients with thymic malignancies. We reviewed charts of patients diagnosed with epithelial thymic malignancies at University of Florida between 2001 and 2021. Forty‐seven patients were included in the final analysis, including 40 patients with thymoma and seven patients with thymic carcinoma. Regarding thymomas, median age at clinical presentation was in the sixth decade of life, consistent with other studies. Several investigations have demonstrated a bias for male or female sex as well as no predilection; in this study, no significant difference in incidence was noted between genders.^6^, ^7^, ^8^, ^9^, ^10^, ^11^ Relative frequencies of thymoma subtype in this study were A (7.5%), AB (22.5%), B1 (25.0%), B2 (20.0%), and B3 (15.0%), similar to those observed in the literature with subtype A (11%), AB (23%), B1 (17%), and B2 (28%), and B3 (21%).^7^

The most significant observed difference between thymoma subtypes on presentation is age at diagnosis. Thymoma subtypes A and AB have been reported to manifest in higher ages than other subtypes. In our cohort, patients with subtypes A and AB presented at significantly higher ages (mean of 71 and 63 years, respectively) when compared to the next highest age (mean of 52 years in subtype B2). The impact of WHO histological subtype on prognosis has been less clear. In the largest analysis of thymomas, there was no significant association between subtype and outcome.^7^ Indeed, thymoma subtypes A and AB have more often been cited for their benign nature, although other studies have indicated an equally malignant potential, demonstrating insufficient correlation between subtype and behavior.^12^, ^13^, ^14^, ^15^, ^16^, ^17^ In this study, patients with thymoma subtype AB were most likely to present with early‐stage disease. Moreover, rates of recurrence were highest in patients with thymoma subtypes B2 and B3, as observed in other studies.^9^, ^18^, ^19^, ^20^ It must be noted that age of presentation certainly contributes to recurrence rate, as a weaker correlation between thymoma subtypes and recurrence has been demonstrated when matching for age of onset.^7^ Notably, there was no significant relationship between thymoma subtype and overall survival. There is ultimately insufficient evidence regarding the impact of thymoma histological subtype and overall outcomes, justifying the need for long‐term clinical surveillance in all cases.

Due to the rarity of thymomas, prognostic information is limited. A few investigations have independently identified complete resectability and stage as factors significantly associated with mortality in patients with thymomas as well as thymic carcinoma.^21^, ^22^, ^23^, ^24^ A brief review of available published data on thymomas is available in Table 7; this summary is limited to retrospective studies with at least 40 cases detailing prognostic factors and significant associations among individuals with thymomas of any subtype. In our cohort, stage on presentation was similarly associated with increased mortality; however, complete resection was not significantly associated with improved mortality. Several additional prognostic factors were identified within this study. Female sex was associated with increased mortality from thymoma of any subtype, with a mean overall survival of roughly 5 years when compared to over 8 years in males. Contemporary data has suggested that the expression of estrogen receptors in thoracic malignancies may contribute to the gender differential in regard to survivorship.^25^ Briefly, estrogens primarily exert their functions through regulated transcriptional processes downstream of estrogen receptor (ER) subtypes ERα and ERβ. There is conflicting literature on the prognostic effect of estrogen and estrogen receptor expression in thoracic malignancies, including esophageal, lung, and thymus cancers. Some studies have suggested thymic tumor ERα immunoreactivity is correlated with reduced tumor size, clinical stage, and improved overall outcomes; high expression of ERβ in thymic tumors and a significant association with clinical stage have also been observed. The underlying mechanism of estrogen expression in thymic malignancies is clearly complex and warrants further evaluation.

**TABLE 7:** Review of retrospective studies detailing prognostic data in individuals with thymomas

Study	Country	Type	Number of patients	Significant associations and prognostic factors
Weis et al.^7^	Multicenter	International database	4221	Increasing age, higher stage, and resection status impacts survival and recurrence. Histology impacts only recurrence
Alothaimeen et al.^8^	Saudi Arabia	Single institution	56	Lower Masaoka staging and non‐thymoma histology are associated with extended overall survival
Ruffini et al.^9^	Multicenter	International database	2151	Masaoka stages III–IV, incomplete resection and non‐thymoma histology are associated with higher recurrence and worsened survival
Gripp et al.^10^	Germany	Single institution	70	Stage, histology, and Karnofsky performance status, predict overall survival. Late‐stage relapses are significantly reduced by postoperative radiotherapy
Rea et al.^13^	Italy	Single institution	132	Radical resection, Masaoka clinical staging, tumor histologic subtype and resectable tumor recurrence are significant prognostic factors
Wright et al.^15^	United States	Single institution	179	Size of 8 cm or larger is an independent risk factor regardless of stage. Recurrence rates are significantly correlated with tumor subtypes B2, B3, and C
Kondo et al.^16^	Japan	Multiple institutions	100	Significant differences in disease‐free survival exist between subtypes A and AB vs. B1 and B2 as well as between subtype B3 versus C
Jain et al.^17^	United States	Single institution	500	Significant difference in survival exists for patients with tumor subtypes A and AB with and without recurrence, showing poor correlation with tumor behavior
Rena et al.^18^	Italy	Single institution	178	Masaoka staging, tumor histologic class, and resection status are significant prognostic factors, whereas age‐ and sex‐associated myasthenia gravis are not
Margaritora et al.^19^	Italy	Single institution	317	Myasthenia gravis concomitance does not significantly impact operative outcome, but prolongs long‐term survival and disease‐free survival
Ríos et al.^20^	Spain	Single institution	44	Clinical stage and histological subtype are significant prognostic factors. Surgical technique on resection may affect survival, but not on multivariate analysis
Kamata et al.^21^	Japan	Single institution	60	Incomplete resection is associated with significantly poorer prognosis. Masaoka staging significantly impacts overall survival
Kim et al.^22^	South Korea	Single institution	108	Tumor subtype B3 has intermediate prognostic impact; subtypes A‐B2 versus B3 versus C predicts survival. Masaoka staging is the most important prognostic factor
Shinohara et al.^23^	Japan	Single institution	54	Complete macroscopic resection is a positive prognostic factor, even in higher‐stage presentations
Zhang et al.^26^	China	Single institution	104	Patients with myasthenia gravis, Masaoka stage III–IV and tumor subtype B2 or B3 have a poorer prognosis
Zhai et al.^27^	China	Single institution	470	No differences in progression‐free, distant metastasis‐free, and local‐regional relapse‐free survival between patients with and without myasthenia gravis
Vachlas et al.^28^	Greece	Single institution	79	Patients with myasthenia gravis more often present with tumor subtypes B2 and B3. Partial or complete myasthenia response is a significant prognostic factor

The presence of concomitant autoimmune disease, namely myasthenia gravis, has also been proposed as a negative prognostic factor for thymomas. However, several studies, including a large investigation in 2020, have reported no significant association between the presence of myasthenia gravis and overall outcomes in patients with thymoma.^26^, ^27^, ^28^ Myasthenia gravis in thymomas has demonstrated no sex predilection and worsened severity of disease when compared to non‐thymoma myasthenia gravis.^29^ Therefore, it has been suggested that symptomatic myasthenia gravis may inadvertently promote earlier detection of underlying malignancies, including thymomas. In this study, the presence of myasthenia gravis was not associated with increased mortality but did demonstrate a significant association with higher rates of tumor recurrence. One study investigated the presence of autoantibodies in addition to anti‐ acetylcholine receptor antibodies in patients with thymoma and myasthenia gravis.^30^ Results indicated a higher and similar prevalence of titin autoantibodies in both late‐onset myasthenia gravis and thymoma myasthenia gravis when compared to early‐onset non‐thymoma myasthenia gravis; however, the implications of these findings on malignancy behavior and recurrence potential is not yet understood.

Identification of risk factors significantly associated with onset of epithelial thymic cancer has also lacked clarity. Previously published literature has suggested that there are no known risk factors for developing thymic cancer. However, in 2019, Eriksson and colleagues investigated risk factors in 103 patients with thymoma when compared to population controls and reported that tobacco use was moderately related with diagnoses of thymoma (odds ratio 1.4, 95% confidence interval 0.9–2.2).^31^ Over half of patients in our cohort had a history of smoking. While there was not an appropriate control population to allow demonstration of significant association, this striking level of tobacco use is noteworthy.

Data demonstrating the efficacy of various treatment modalities of thymomas and thymic carcinoma has been insufficient. Surgical resection remains the mainstay of treatment for epithelial thymic malignancies. All patients with thymoma in this study received surgical resection except one individual, who did not receive any treatment. The significance of postoperative radiotherapy and chemotherapy in thymoma remains controversial due to paucity of data.^32^, ^33^ Generally, surgical margins and pathologic staging are used to guide postoperative management.^34^ Rimner et al., which is by far the largest study, found survival benefit with postoperative radiotherapy in stage II and III thymoma.^35^ Survival benefits were found in all histological subtypes, especially thymoma subtypes B1, B2, and B3. In our study, nine patients received radiotherapy (2/3 with subtype A, 1/8 with subtype B2, and 6/6 with subtype B3), which may explain higher recurrence in patients with B2 subtype compared to those with B3 subtype thymomas, where all patients received radiation. Overall, the use of chemotherapy and radiotherapy did not significantly impact survival in this study.

Thymic carcinoma is a more rare, understudied, and aggressive form of epithelial thymic malignancy. In our cohort, patients with thymic carcinoma presented with more advanced disease, which is consistent with the literature.^10^ Four patients had stage IV disease, of which three died within 18 months due to disease progression. Four of seven patients received surgery; these patients had an overall better survival. Five of seven patients received platinum‐based chemotherapy at some point during treatment. One patient received salvage nivolumab, which was switched to pembrolizumab after further disease progression. The role of immune checkpoint inhibitors in the treatment of thymoma and thymic carcinoma is not well defined. These tumors tend to have relatively few mutations, but have demonstrated high programmed death‐ligand 1 expression and thus have been theorized to be good candidates for immunotherapy.^36^ Results of immune checkpoint inhibitor use have been mixed. There is some evidence that immune checkpoint inhibitors may improve outcomes in patients with epithelial thymic malignancies; however, there appears to be higher rates of immune‐related adverse events in these tumors than in other cancers.^36^, ^37^, ^38^ One non‐randomized phase II study of 10 patients with thymic carcinoma treated with pembrolizumab demonstrated a response rate of 22.5% and progression‐free survival of 4.2 months.^39^ Conversely, a single‐armed trial of 15 patients with thymic carcinoma treated with nivolumab showed no positive response.^40^

Our study has several limitations. A large time‐span of 20 years was necessary to achieve even a modest sample size, and the retrospective nature of the data gathered limits the power of the associations we identified. For instance, while an association between myasthenia gravis and increased mortality was not demonstrated, this study is far too small to elicit definitive conclusions regarding this relationship; results should be interpreted with caution. Importantly, the cause of death in patients within this study was not documented. Of nine total patients with thymoma who died, including seven female patients, only one died within a year of diagnosis. Thus, no significant inferences regarding the impact of onset of thymoma and long‐term outcomes can be established.

## AUTHOR CONTRIBUTIONS

**Ellery Altshuler:** Conceptualization (lead); formal analysis (lead); investigation (lead); methodology (lead); writing – original draft (lead); writing – review and editing (lead). **Akash Mathavan:** Conceptualization (equal); investigation (equal); methodology (equal); writing – original draft (equal); writing – review and editing (equal). **Akshay Mathavan:** Conceptualization (equal); investigation (equal); methodology (equal); writing – original draft (equal); writing – review and editing (equal). **Urszula Krekora:** Investigation (equal); methodology (equal); writing – original draft (equal); writing – review and editing (equal). **Mohit Mathavan:** Investigation (equal); methodology (equal); writing – original draft (equal); writing – review and editing (equal). **Keegan Hones:** Investigation (equal); methodology (equal); writing – original draft (equal); writing – review and editing (equal). **Karen Daily:** Supervision (lead); writing – original draft (equal); writing – review and editing (equal).

## CONFLICT OF INTEREST

The authors have stated explicitly that there are no conflicts of interest in connection with this article.

## ETHICS STATEMENT

Approval for this study was obtained from the University of Florida Institutional Review Board (IRB202102647).

## Supporting information


**Appendix S1:** Supporting Information

## Data Availability

All data generated or analyzed during this study are included in this published article and referenced articles are listed in the References section. Additional information, including survival curve figures, can be found in Supporting information.
