# Supplementary material for: Clinical characteristics, prognostic factors, and long‐term outcomes associated with epithelial malignancies of the thymus: A 20‐year single‐institution experience
Source: Cancer Rep (Hoboken). 2022 Nov 12;6(3):e1750. doi: 10.1002/cnr2.1750 (PMC10026295; doi:10.1002/cnr2.1750)
Supplement: Supplementary file 1 — Appendix S1: Supporting Information [file CNR2-6-e1750-s001.docx]

**Supporting Information for**

***Clinical characteristics, prognostic factors, and long-term outcomes associated with epithelial malignancies of the thymus: A twenty-year single-institution experience***

Ellery Altshuler^1^, Akash Mathavan^2^, Akshay Mathavan^2^, Urszula Krekora^3^, Mohit Mathavan^4^, Keegan Hones^2^, Karen Daily^1,5^

^1^Department of Internal Medicine, University of Florida, Gainesville, FL

^2^University of Florida College of Medicine, University of Florida, Gainesville, FL

^3^University of Central Florida College of Medicine, University of Central Florida, Orlando, FL

^4^Department of Internal Medicine, St George’s University School of Medicine, Great River, New York

^5^Division of Hematology and Oncology, University of Florida, Gainesville, FL


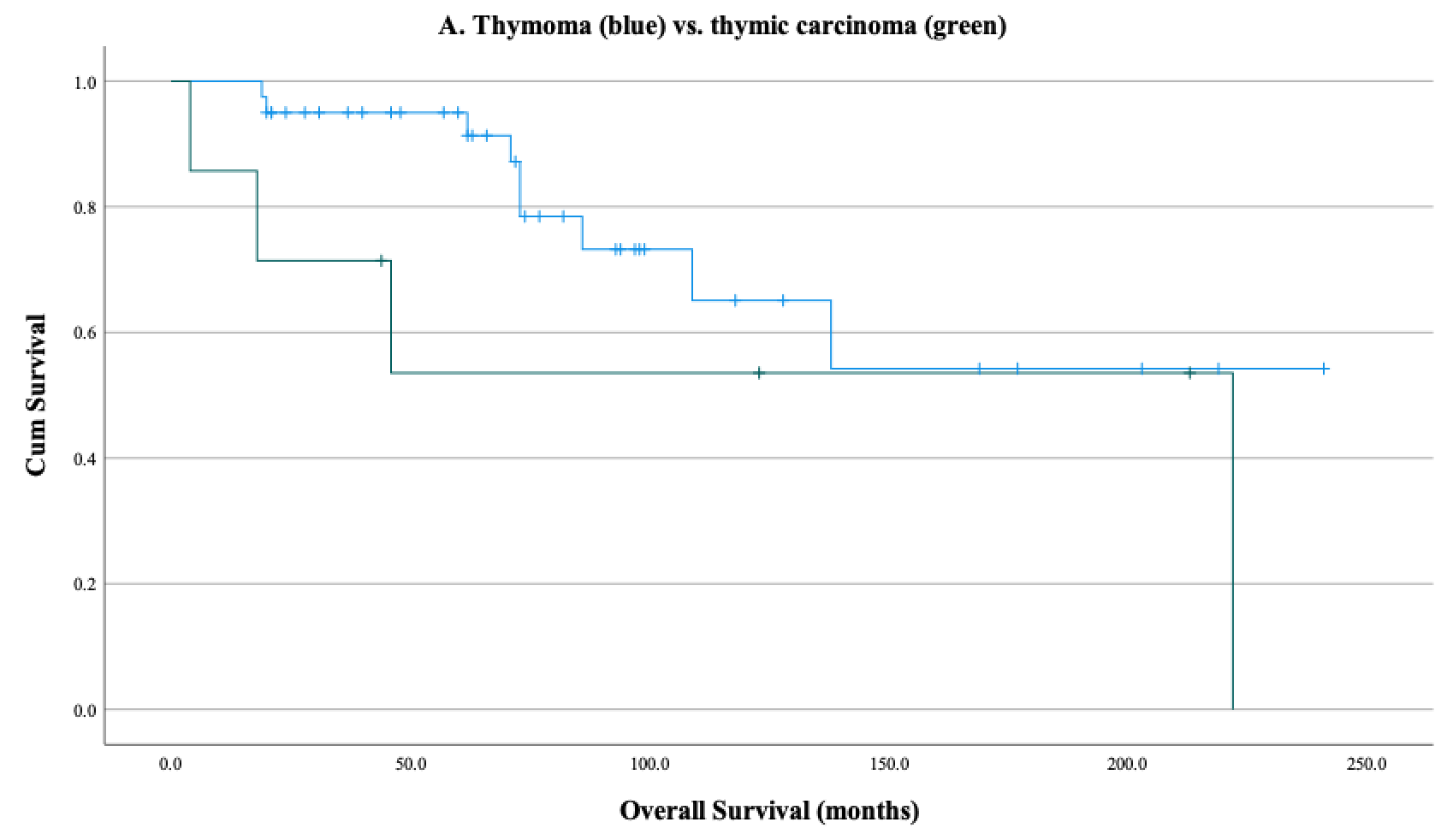


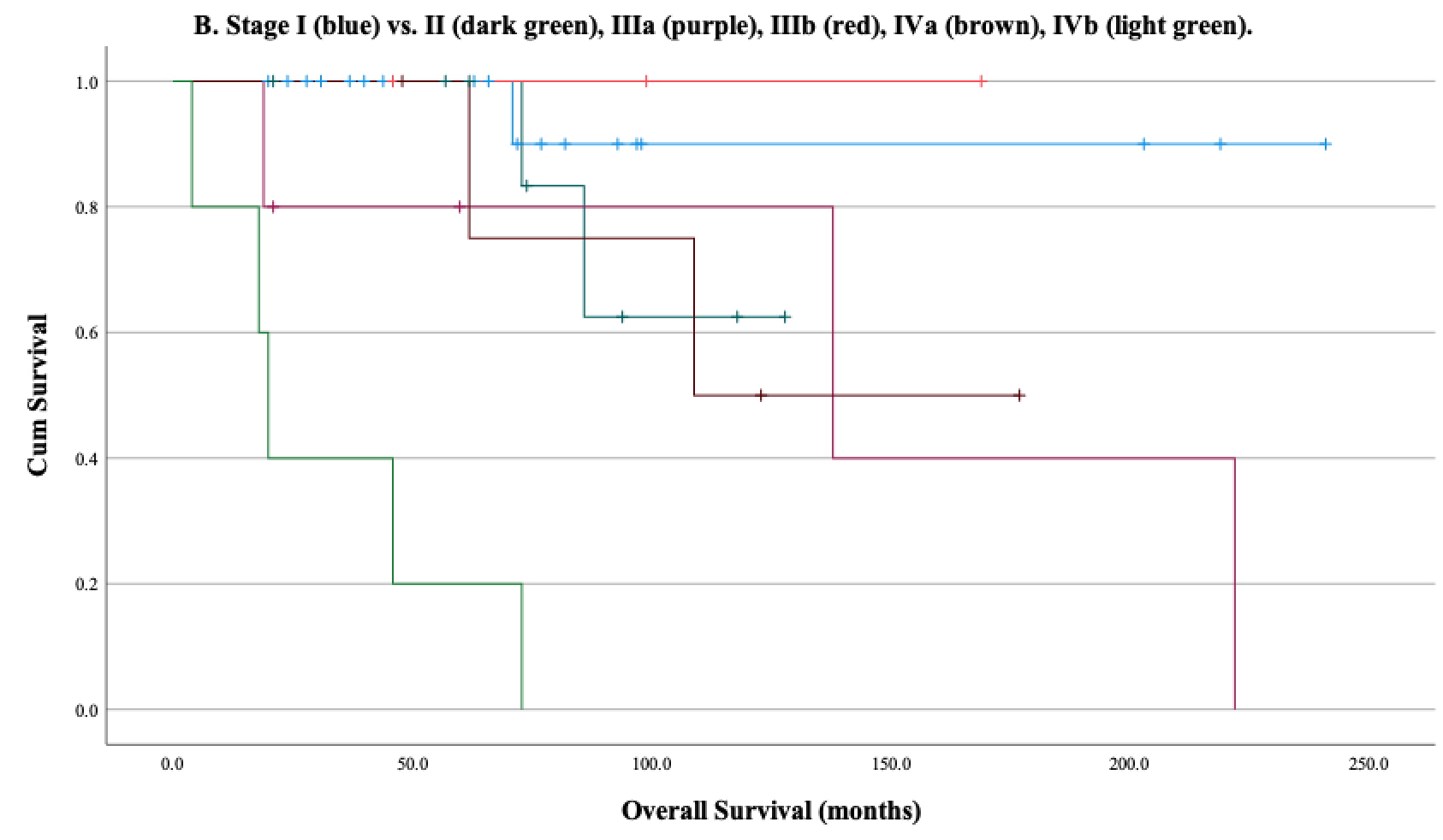


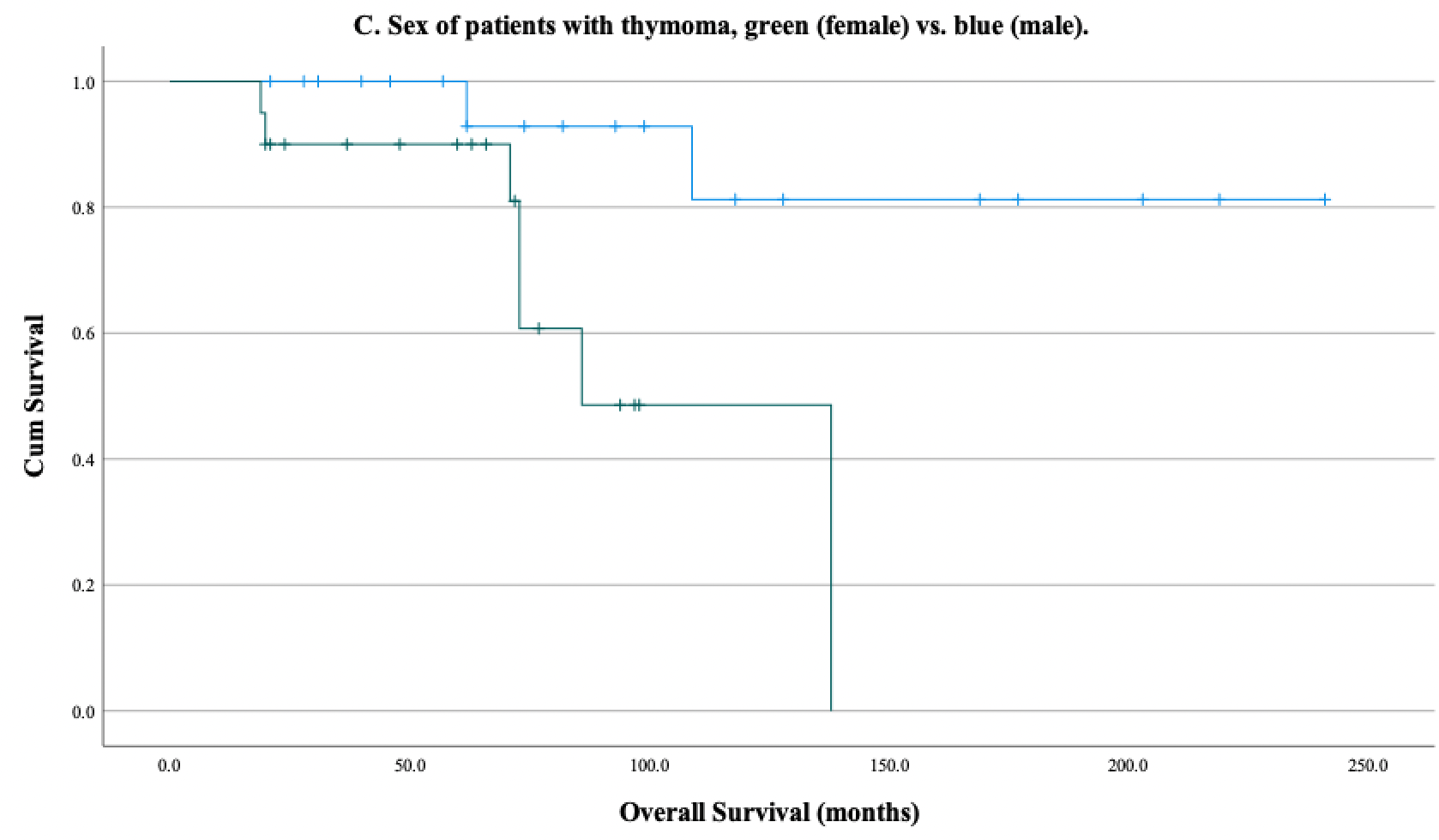


A: Kaplan-Meier survival estimate for patients with thymoma (blue) and thymic carcinoma (green). B: Patient survival curves based on AJCC staging. Stage I is depicted in blue, stage II in dark green, stage IIIa in purple, stage IIIb in red, stage IVa in brown, and stage IVb in light green. C: Survival curves for patients with thymoma based on sex depicted as female (green) and male (blue). Log-rank (Mantel-Cox) significant at p = 0.008.
